# In vitro injection of osteoporotic cadaveric femurs with a triphasic calcium‐based implant confers immediate biomechanical integrity

**DOI:** 10.1002/jor.24239

**Published:** 2019-03-20

**Authors:** John D. Stroncek, Jonathan L. Shaul, Dominique Favell, Ronald S. Hill, Bryan M. Huber, James G. Howe, Mary L. Bouxsein

**Affiliations:** ^1^ AgNovos Healthcare 7301 Calhoun Place Suite 100 Rockville Maryland 20855; ^2^ Copley Hospital 528 Washington Hwy Morrisville Vermont 05661; ^3^ Center for Advanced Orthopedic Studies, Beth Israel Deaconess Medical Center and Dept. of Orthopedic Surgery Harvard Medical School 330 Brookline Ave Boston Massachusetts 02215

**Keywords:** bone mechanics and finite element analysis, bone tissue engineering and repair, bone aging

## Abstract

Current pharmaceutical therapies can reduce hip fractures by up to 50%, but compliance to treatment is low and therapies take up to 18 months to reduce risk. Thus, alternative or complementary approaches to reduce the risk of hip fracture are needed. The AGN1 local osteo‐enhancement procedure (LOEP) is one such alternative approach, as it is designed to locally replace bone lost due to osteoporosis and provide immediate biomechanical benefit. This in vitro study evaluated the initial biomechanical impact of this treatment on human cadaveric femurs. We obtained 45 pairs of cadaveric femurs from women aged 77.8 ± 8.8 years. One femur of each pair was treated, while the contralateral femur served as an untreated control. Treatment included debridement, irrigation/suction, and injection of a triphasic calcium‐based implant (AGN1). Mechanical testing of the femora was performed in a sideways fall configuration 24 h after treatment. Of the 45 pairs, 4 had normal, 16 osteopenic, and 25 osteoporotic BMD T‐scores. Altogether, treatment increased failure load on average by 20.5% (*p* < 0.0001). In the subset of osteoporotic femurs, treatment increased failure load by 26% and work to failure by 45% (*p* < 0.01 for both). Treatment did not significantly affect stiffness in any group. These findings provide evidence that local delivery of the triphasic calcium‐based implant in the proximal femur is technically feasible and provides immediate biomechanical benefit. Our results provide strong rationale for additional studies investigating the utility of this approach for reducing the risk of hip fracture. © 2019 The Authors. *Journal of Orthopaedic Research*® Published by Wiley Periodicals, Inc. on behalf of Orthopaedic Research Society.

Osteoporosis, characterized by compromised bone strength leading to an increased fracture risk, impacts over 200 million women worldwide.^1^, ^2^ Hip fractures are the most devastating fractures, leading to increased mortality and morbidity. There were 1.6 million hip fractures worldwide in 2000^3^ and this number is projected to increase to 2.6 million by 2025.^4^ Mortality of hip fracture patients can be up to 25% within a year^5^, ^6^, ^7^ and the risk for a second hip fracture is highest in the first year following the occurrence of an initial fracture.^8^

The vast majority of hip fractures occur subsequent to a fall, particularly a fall to the side with impact to the hip.^9^ While currently available pharmaceutical treatments improve proximal femur bone mineral density (BMD), they can take up to 18 months to significantly reduce the risk of fracture.^10^ Available pharmaceutical therapies provide up to an approximately 50% reduction in hip fractures,^11^, ^12^ but patient compliance is about 50% after 1 year.^13^, ^14^ Anabolic therapies, such as teriparatide, abaloparatide, and romozosumab, stimulate new bone formation and can lead to significant increases in BMD.^15^, ^16^, ^17^ However, these medications are delivered via subcutaneous injection and have patient compliance issues with nearly 90% of patients in the United States discontinuing medication before the 2 year maximum treatment period.^14^ The gap between the initiation of antiresorptive or anabolic osteoporosis therapies, often only prescribed after an initial fragility fracture, and the time to fracture risk reduction with these treatments means that these patients remain at a high risk for a hip fragility fracture. Thus, given the limitations of existing methods, there is a significant need for new approaches to reduce hip fractures.^18^

Alternate surgical approaches that have gained interest recently, in part because they are not dependent on patient compliance, include femoral bone augmentation procedures aimed at increasing femoral strength.^18^ In support of these approaches, biomechanical studies have evaluated femoral augmentation using non‐resorbable polymethyl methacrylate (PMMA),^19^, ^20^, ^21^, ^22^, ^23^ silicone rubber,^24^, ^25^ or prophylactic hardware.^26^, ^27^ Although these techniques have shown some promise to immediately improve femoral strength, inherent limitations have hindered their clinical adoption. Ideally, a surgical procedure should be minimally invasive and safe. The intervention must not increase the immediate risk of hip fracture and must also provide long‐term fracture risk reduction, and if a subsequent fracture does occur, the intervention should not interfere with a standard approach for fracture repair.

This study evaluated a new minimally invasive approach intended to strengthen the hip by addressing local osteoporotic bone loss by delivering a resorbable, triphasic calcium sulfate/calcium phosphate implant material (AGN1) to the proximal femur. The implant material sets in situ and is designed to be resorbed and replaced with new bone to improve femoral strength and reduce hip fracture risk in osteoporotic patients. The aim of this study was to evaluate the immediate effect of AGN1 delivered using this local osteo‐enhancement procedure (LOEP) on the mechanical strength of cadaveric femurs in a sideways fall configuration. We hypothesized that this approach could deliver the implant material without compromising the biomechanical properties of the proximal femur.

## METHODS

### Specimen Preparation

Forty‐six pairs of fresh frozen human cadaveric femurs consented for research use from women greater than 60 years of age were obtained from commercial tissue banks. One pair of femurs from a donor with a history of bone cancer was excluded from the study. One femur from each pair was randomly assigned to either the treated or control group. The treated group was injected with the triphasic calcium sulfate/calcium phosphate self‐setting implant material and the contralateral femurs were left as the untreated control group.

Specimens were prepared for imaging, treatment, and mechanical testing by first cutting the femoral diaphysis distal to the lesser trochanter at a distance equal to three‐times the femoral midshaft diameter. To ensure consistent alignment for imaging and testing, the samples were potted within a test fixture. Adduction and internal rotation were set to 10° and 15°, respectively, to mimic a sideways fall configuration. Femurs were fixed with set screws applied radially around the shaft of each femur and then urethane was poured to secure the positioning.

### Bone Mineral Density

Areal bone mineral density (aBMD g/cm^2^) of the proximal femur, including total hip and femoral neck regions, were assessed by dual‐energy X‐ray absorptiometry (DXA) (GE Lunar Prodigy, Madison, WI). Femurs were immersed in a container of rice to simulate the presence of soft tissue^28^ and then scanned with the femoral neck axis parallel to the scanner table.

### AGN1 Triphasic Implant Material

AGN1 is a triphasic implant material consisting of calcium sulfate, brushite, and β‐tricalcium phosphate (AgNovos Healthcare, Rockville, MD). AGN1 sets through the hydration of calcium sulfate hemihydrate to calcium sulfate dihydrate. This exothermic reaction does not exceed 35°C and is complete within 60 min after mixing. Brushite is distributed throughout the calcium sulfate and provides structural integrity to the implant material as it resorbs. β‐tricalcium phosphate is present as granules (approximately 250 μm diameter) distributed throughout the set implant material and provides niduses for new bone formation.

### AGN1 Injection of the Proximal Femur

**Figure 1:**
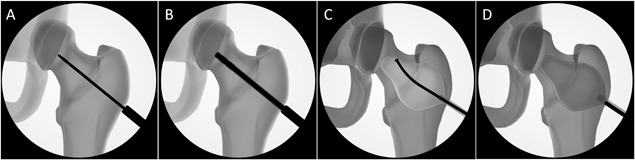
A rendering of AGN1 injection procedure into the proximal femur. A 2.5 mm guide pin was inserted into the femoral neck (A), a 5.3 mm cannulated drill was inserted over the guide pin (B), the implant site was manually debrided to loosen fat and marrow (C) which was removed with irrigation and suction, and the implant material was injected into the proximal femur (D).

The femur from each pair assigned to the treated group was injected with AGN1 as follows. Using fluoroscopy, a 2.5 mm guide pin was inserted through the lateral femoral cortex, approximately 2 cm distal to the greater trochanter, and then through the intertrochanteric region into the femoral neck (Fig. 1A). A cannulated drill over the guide pin was used to create a 5.3 mm portal through the cortex which was then extended from the lateral subtrochanteric region through the femoral neck (Fig. 1B). Using a blunt probe debrider, the proximal femur was manually debrided to loosen fat and non‐structural marrow elements, which were removed via irrigation and suction (Fig. 1C). The triphasic calcium sulfate/calcium phosphate implant material was then manually injected into the proximal femur (Fig. 1D) using a force on average of 113 ± 19 N (mean ± SD). The injection began at the apex of the enhancement site with the injection cannula systematically moving toward the lateral access portal. Note that with the open cortical portal and the connection to the femoral medullary canal, no increase in intra‐femoral pressure would be expected. The volume of material injected into each defect was recorded. All femurs were kept at room temperature for several hours to complete post‐injection computed tomography (CT) imaging ensuring that the AGN1 was completely set before transfer to 4°C. Femurs were stored for approximately 24 h prior to mechanical testing. Femurs were warmed to room temperature before testing the next day.

### Computed Tomography Imaging of Femurs

Distribution of implant material after injection was evaluated using computed tomography (CT) scans of each specimen prior to and after the treatment procedure using a 16 slice scanner (Brightspeed, General Electric, Fairfield, CT) with a slice thickness/spacing of approximately 0.625 mm. All scans were acquired at 120 kVP using a pitch of 0.93 and were reconstructed using the standard bone algorithm. Scans were reconstructed with small (420 mm diameter) and large (500 mm diameter) field of view reconstructions. For the small field of view reconstructions, the smallest reconstruction diameter able to capture the largest femur was used and kept constant for all cadavers.

### Mechanical Testing in Sideways Fall Configuration

**Figure 2:**
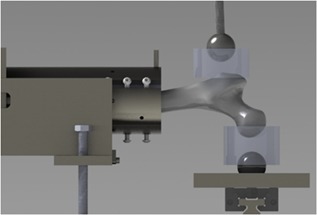
Schematic of mechanical testing set up.

Both treated and control femurs were tested to failure in a sideways fall configuration, as previously reported.^29^ In brief, the greater trochanter and femoral head were partially embedded in urethane as described above, which was custom fit for each specimen. Mechanical testing was performed using a servohydraulic load frame (MTS Systems 810, Eden Prairie, MN). Compressive force was applied to the greater trochanter (Fig. 2) at a constant rate of 100 mm/s. Load and displacement data were acquired at 1000 Hz, and tests were recorded using a high‐speed video system (Fastec T53‐100S, San Diego, CA). The force and displacement data were used to assess stiffness, failure load (defined as the maximum load), and work to failure.

### Statistical Analysis

Power analyses were performed prior to the study using published data,^30^ sizing the study to demonstrate the procedure does not diminish femoral bone strength by more than 5% and to confirm that the initial procedure would not increase the risk of fracture by compromising femoral strength. We computed standard descriptive statistics and evaluated all data for outliers. Paired Student's *T*‐tests were used to compare outcomes between treated and control femurs. We also performed analyses after stratifying groups by osteoporosis status based on femoral neck DXA BMD T‐scores. Normal, osteopenic, and osteoporotic were defined as a femoral neck BMD T‐score > −1, −1 to −2.5, and <−2.5, respectively. Differences in donor demographics and injection volumes between BMD groups were evaluated using a one‐way ANOVA followed by a Tukey post hoc test. All analyses were performed using Minitab Version 18. *p*‐values ≤0.05 were considered statistically significant. Data are presented as mean ± standard deviation, unless otherwise noted.

## RESULTS

Average donor age was 77.8 ± 8.8 years (range: 60–96 years) with a BMI of 27.0 ± 9.7 kg/m^2^ (range: 12–51 kg/m^2^) (Table 1). The average femoral neck BMD was 0.651 ± 0.182 g/cm^2^, corresponding to a T‐score of −2.8 ± 1.3; average total hip BMD was 0.674 ± 0.176 g/cm^2^, corresponding to a T‐score of −2.6 ± 1.3. Of the 45 femoral pairs, 4 had normal, 16 osteopenic, and 25 osteoporotic femoral neck T‐scores. Donors with normal femoral neck BMD T‐scores tended to be younger and weigh more than the osteopenic or osteoporotic donors (Table 1).

**Table 1:** Demographic Characteristics of Donors and Femoral Specimens (mean ± SD)

Parameter	All Donors	Normal	Osteopenic	Osteoporotic
Number of Paired Femurs	45	4	16	25
Age (Years)	77.8 ± 8.8	71.3 ± 4.5	75.0 ± 7.7	80.7 ± 8.9
Height (cm)	160.7 ± 8.7	165.7 ± 9.4	161.2 ± 9.4	159.5 ± 8.1
Weight (kg)	70.3 ± 27.5	102.0 ± 14.7	76.7 ± 22.6	61.1 ± 27.5^a^
Body Mass Index (kg/m^2^)	27.0 ± 9.7	37.5 ± 6.1	29.4 ± 7.9	23.8 ± 9.9^a^
Femoral Neck T‐Score	−2.8 ± 1.3	−0.2 ± 0.7	−2.0 ± 0.4	−3.7 ± 0.7

^a^ *p* ≤ 0.05 vs. normal femurs.

Prior to AGN1 injection, femoral neck BMD did not differ between the treated and control femurs (*p* = 0.94) (Table 2) nor did total hip BMD (data not shown). Femoral neck BMD also did not differ between pretreatment treated and control femurs within the normal, osteopenic, or osteoporotic subgroups (Table 2) nor did total hip pretreatment BMD differ between these groups (data not shown).

**Table 2:** Pretreatment Femoral Neck BMD (g/cm^2^) (mean ± SD)

Group	Control	Treated	*p*‐Value
All Femurs (*n* = 45)	0.651 ± 0.188	0.652 ± 0.190	0.94
Normal (*n* = 4)	1.036 ± 0.137	0.984 ± 0.078	0.35
Osteopenic (*n* = 16)	0.754 ± 0.066	0.781 ± 0.087	0.26
Osteoporotic (*n* = 25)	0.523 ± 0.103	0.515 ± 0.107	0.71

**Figure 3:**
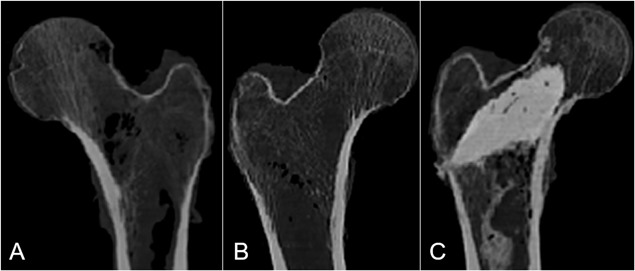
Pre‐ and post‐treatment CT images of osteopenic paired femurs. Control (A), Treated before debridement and AGN1 injection (B), and after AGN1 injection (C).

**Figure 4:**
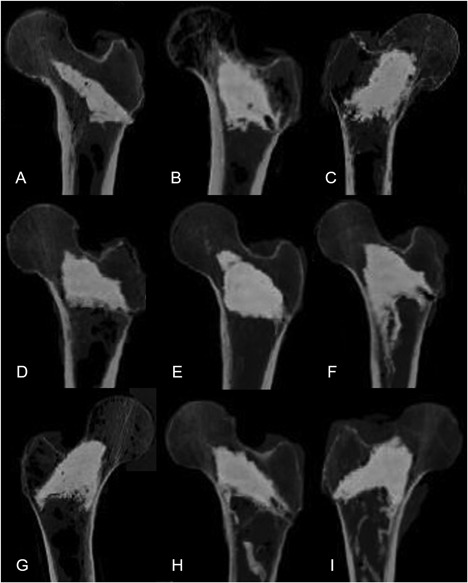
AGN1 distribution in cadaveric femurs following treatment. Normal (A and B), Osteopenic (C and D), and Osteoporotic (E to I) femurs.

The average volume of AGN1 injected into the treated femurs was 19 ± 4 cc (range: 11–26 cc). Stratification of specimens by femoral neck BMD T‐score showed that the injected volume of AGN1 was greater in osteopenic and osteoporotic femurs compared to normal femurs: 19 ± 4 cc in osteopenic femurs, 20 ± 4 cc in osteoporotic femurs, and 13 ± 5 cc in normal femurs (*p* < 0.005). CT imaging after the procedure showed consistent AGN1 distribution within the femoral neck and intertrochanteric region, extending toward the lateral cortical wall (Figs. 3 and 4).

**Figure 5:**
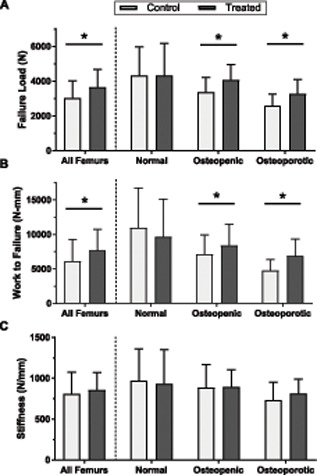
Failure load (A), work to failure (B), and stiffness (C) of all femurs and femurs stratified by femoral neck BMD T‐score: Osteoporotic (T‐score < −2.5), osteopenic (−1 to −2.5), and normal (T‐score > −1). Data presented as mean ± standard deviation; *Indicates *p* < 0.05 versus the paired control.

For all femurs combined, failure load of treated femurs was on average 20.5% higher than the contralateral untreated control femurs (Fig. 5) (*p* < 0.001). Work to failure of all combined treated femurs was also significantly higher after treatment than that of the contralateral matched control femurs (*p* < 0.001). Stiffness was not statistically different between groups (*p* = 0.29).

Failure load in treated femurs was also significantly greater than that of control femurs in the osteopenic (4061 ± 896 vs. 3368 ± 845 N; *p* < 0.001) and the osteoporotic groups (3264 ± 823 vs. 2591 ± 669 N; *p* < 0.001) (Fig. 5A). Failure load was numerically higher in 88% (14 of 16) of osteopenic and 84% (21 of 25) of osteoporotic treated femurs compared to untreated contralateral control. Treatment had no influence on failure load in femurs with BMD T‐scores in the normal range (*p* = 0.97) (Fig. 5A). Work to failure was significantly higher in treated compared to control in both osteopenic (8392 ± 3075 vs. 7150 ± 2797 N‐mm; *p* < 0.05) and osteoporotic femurs (6894 ± 2453 vs. 4766 ± 1565 N‐mm; *p* < 0.001), but not in femurs with BMD T‐scores in the normal range (Fig. 5B). Work to failure was higher in 69% (11 of 16) of osteopenic and 88% (22 of 25) of osteoporotic treated femurs compared to the untreated contralateral control. There was no correlation between injection volume and changes in failure load (*r* ^2^ = 0.016, ns). Treatment did not significantly change femoral stiffness in any group (Fig. 5C).

Video analysis of the mechanical testing of 83 femurs (7 lacked video) demonstrated that fractures were either intertrochanteric (70.7% treated, 78.6% control) or femoral neck (29.3% treated, 21.4% control) fractures. The distribution of fracture location was similar among treated and control femurs. In the treated group, none of the fractures occured through the lateral cortical access portal.

## DISCUSSION

In this in vitro study, we assessed the initial biomechanical impact of treating human cadaveric proximal femurs from female donors over 60 years of age with a triphasic calcium sulfate/calcium phosphate implant material. We demonstrated that this procedure is technically feasible and does not adversely affect the biomechanical properties of the proximal femur in a simulated sideways fall immediately after treatment. In fact, the failure load and work to failure were significantly greater in treated osteopenic and osteoporotic femurs compared to control contralateral femurs. No fractures occurred through the lateral cortical access portal, providing further evidence that the procedure has no deleterious effects on femoral strength.

The positive effects of the procedure were greatest in the osteoporotic femurs, where failure load and work to failure increased by 26% and 45%, respectively. Though not directly comparable, the increase in failure load is larger than that reported from finite element analysis of CT scans of the proximal femur in randomized clinical trials of osteoporosis therapies.^31^, ^32^, ^33^, ^34^, ^35^ In particular, compared to postmenopausal women treated with placebo, those treated with denosumab had 5% and 9% higher femoral failure load at 12 and 36 months^33^ which translated to a 40% to 50% reduction in hip fracture risk at 36 months.^11^, ^36^ This observation suggests the increases in femoral strength observed in this study immediately after injection of the implant material would be clinically relevant.

Based on a prospective epidemiologic study of hip fracture risk, Kopperdahl et al.^37^ proposed a threshold of 2,900 N for “fragile bone strength” of female proximal femurs. In the osteoporotic femurs in this study, only 28% (7/25) of control femurs had a failure load greater than 2,900 N whereas 72% (18 of 25) of treated femurs exceeded the “fragile bone strength” threshold of 2,900 N. These data infer that the positive strength increment observed immediately after implant injection is likely to be clinically important and has the potential to reduce fracture risk.

Previous efforts to prevent hip fractures using mechanical methods, reviewed in Varga et al.,^38^ included prophylactic pinning with metal implants^27^, ^39^ or a PEEK rod system,^20^, ^26^ and injecting silicone rubber^24^, ^25^ or PMMA.^19^, ^21^, ^22^, ^23^, ^40^ The metal systems in general failed to improve biomechanical properties and introduced concerns about stress risers.^38^ Using silicone rubber to fill the proximal femur failed to improve biomechanics.^24^, ^25^ Femoral augmentation studies using large volumes of PMMA (30–40 cc) injected into the proximal femur reported up to an 80% increase in femoral failure load and up to a 188% increase in energy absorption.^19^, ^20^, ^40^ However, due to the 29°C exothermic temperature increase above baseline temperatures during setting and the resultant concerns about thermally‐induced tissue necrosis, the technique has not been pursued clinically. Studies using reduced volumes of PMMA reported inconsistent results. For instance Beckmann et al.^22^ evaluated four femoral augmentation techniques with differing placement of the cement, and found significant increases in failure load and energy to fracture for a sideways fall testing configuration, even with minimal cement volumes. In contrast, another study found that 15 cc of PMMA implanted into either the femoral neck or intertrochanteric region did not increase biomechanical properties.^21^ Another technique implanted PMMA (mean volume 10.8 cc) in a “v‐shaped” pattern (i.e., two paths extending from the lateral cortex in the greater trochanter to the superior and inferior aspect of the neck) and found a significant increase in energy to fracture, but no effect on fracture or yield loads.^23^ Notably, Basafa et al.^41^ used finite element analysis to demonstrate that femoroplasty‐based augmentation of the superior aspect of the femoral neck close to the cortex optimized protection against sideways fall fractures. The procedure we tested fills a similar area of the femoral neck with AGN1 and may explain, in part, the positive biomechanical effects observed in the current study. In all cases, PMMA presents challenges for surgical revisions in the event of a fracture and may induce thermal damage. A PEEK Y‐Strut construct that implants two rods (one 9 × 80–100 mm in the proximal femur and one 8 × 55–80 mm down the medullary canal that are then cemented together using PMMA) immediately improved fracture load and energy,^20^, ^26^ but has gained little clinical adoption to date. Based on the above limitations of these procedures, there is a call for new interventional strategies to strengthen the proximal femur.^18^

This minimally invasive procedure to locally deliver AGN1 evaluated in this study offers a new approach to treat osteoporotic bone loss in the proximal femur. In contrast to the above purely mechanical systems, the current method provides immediate biomechanical benefit and a long‐term biological benefit as the implant material is resorbed and replaced with bone. This biological effect was demonstrated in a 12 patient study in postmenopausal osteoporotic women (age 56–83, hip BMD T‐score <−2.5) in which one femur was treated with AGN1 using this minimally invasive technique and the contralateral femur was the control. CT analysis demonstrated that as AGN1 resorbed it was replaced with newly formed bone resulting in a sustained BMD increase of 68% at 1 year, 64% at 2 years, and 57% at 5–7 years.^42^ Finite element analysis estimated that this BMD increase resulted in a 36% increase in femoral strength in a sideways fall configuration in these patients 5–7 years.^42^ These results support the potential long‐term utility of this approach in osteoporotic patients.

The current method is also less invasive than the mechanical systems, as it utilizes a single 5.3 mm drill hole with minimal disruption to the lateral femoral cortex. The location of the access portal was specifically chosen to minimize the potential for creating a clinically significant stress riser. Tran et al.^43^ demonstrated that the subtrochanteric lateral cortex was an optimal access location and did not change the location of fracture when compared to paired controls. Altogether, the current study demonstrated the immediate biomechanical benefits of the procedure in femurs at risk for fragility fractures using an approach that minimizes surgical risk to the treated femur.

Strengths of this study include the large sample size and clinically relevant donor characteristics. Specifically, our study included only female donors over age 60, and more than 90% of the femurs were either osteopenic or osteoporotic by BMD testing. A broad distribution of BMD in femurs allowed for the evaluation of the procedure in varying severity of osteoporosis. Importantly, prior to the start of the procedure there were no differences in the BMD of treated versus control femurs that could contribute to the observed effects in biomechanical properties. The mechanical testing set‐up used is well accepted and a realistic simulation of a sideways fall. In addition, the displacement rate of 100 mm/s results in a peak force at approximately 30 milliseconds that is consistent with a lateral fall on the greater trochanter.^44^, ^45^

The triphasic calcium sulfate/calcium phosphate material utilized in this study is designed to be resorbed and replaced with host bone. Given that the implant material properties will change over time, a limitation of this study is that the findings only relate to the immediate post‐injection period and do not reflect subsequent biologic activity and possible changes in femoral biomechanical properties. Pre‐clinical studies examining the rate and extent of the implant material resorption, bone formation, and mechanical strength post‐implantation are needed to evaluate longer term outcomes of the procedure.

## CONCLUSION

Currently available treatment options do not fully address the needs of patients with osteoporotic bone loss who are at high risk for hip fracture. Results from this study in human cadaveric femurs from women greater than 60 years of age provide evidence that delivering the AGN1 triphasic calcium‐based implant material using a minimally invasive procedure to the proximal femur is technically feasible and provides immediate improvement in biomechanical properties of osteopenic and osteoporotic femurs. These findings provide support for further preclinical and clinical studies investigating this approach to reduce the risk of osteoporotic hip fracture.

## AUTHORS' CONTRIBUTIONS

JGH, BMH, and MLB designed the study. JGH performed the procedures. JDS, JLS, MLB, RSH, and DF prepared the manuscript. RSH, JDS, JLS, and DF analyzed the data. The manuscript was critically revised by RSH and MLB. All authors read, reviewed, and approved the final manuscript.
